# Effects of probiotics in elderly hospitalized tube-fed patients with antibiotics use

**DOI:** 10.1186/s12876-024-03561-9

**Published:** 2024-12-19

**Authors:** Chu-Hsuan Hsia, Hsiu-Yueh Su, Yue-Hwa Chen, Han-Chuan Chuang, Yi-Wen Chien

**Affiliations:** 1 School of Nutrition and Health Sciences, Taipei Medical University, Taipei, 11031 Taiwan, ROC; 2 Department of Dietetics, Taipei Medical University Hospital, Taipei, 11031 Taiwan, ROC; 3 School of Food Safety, Taipei Medical University, Taipei, 11031 Taiwan, ROC; 4 TMU Research Center for Digestive Medicine, Taipei Medical University, Taipei, 11031 Taiwan; 5 Research Center of Food Safety Inspection and Function Development, Taipei Medical University, Taipei, 11031 Taiwan, ROC; 6 Department of Infectious Disease, Taipei Medical University Hospital, Taipei, 11031 Taiwan, ROC; 7 Graduate Institute of Metabolism and Obesity Sciences, Taipei Medical University, Taipei, 11031 Taiwan, ROC; 8 Nutrition Research Center, Taipei Medical University Hospital, Taipei, 11031 Taiwan, ROC; 9 Research Center of Geriatric Nutrition, College of Nutrition, Taipei Medical University, Taipei, 11031 Taiwan, ROC

**Keywords:** Antibiotic-associated diarrhea (AAD), *Clostridium difficile* associated diarrhea (CDAD), Probiotic, Tube feeding elderly, Nutritional status

## Abstract

**Background:**

Several studies revealed the beneficial effects of probiotics against the incidence of antibiotic-associated diarrhea of hospitalized patients but it is rarely to assess the nutrition status. This study investigated the effects of probiotics in elderly hospitalized tube-fed patients with antibiotics use and is the first study that concerns the nutritional status among these patients.

**Methods:**

Elderly hospitalized tube-fed patients who were using antibiotics were recruited. Probiotics were given within 48 h after their first antibiotic therapy, and then twice daily 2 h after consuming antibiotics and a meal; the probiotics were continued to use for an additional 7 days after completion of antibiotics therapy. Anthropometric data, laboratory data, medication records, nutritional status, nutrition intake and data on stool form were collected.

**Results:**

Twenty-nine patients served as probiotic group. 11 patients completed the study in both groups. In probiotic group, the stool form was found to exhibit no significant differences between the beginning and end of antibiotics therapy (5.5 ± 0.8 vs 5.1 ± 1.1, *p* = 0.21), but the stool frequency significantly decreased (2.0 ± 1.0 vs 1.6 ± 0.7, *p* = 0.05). In control group, the stool form between the beginning and end of antibiotics therapy exhibited significant improvement (5.6 ± 1.4 vs 4.5 ± 1.4,* p* = 0.01), but not in the frequency (2.7 ± 2.1 vs 2.4 ± 1.5, *p* = 0.1). The initial NRS 2002 score of the probiotic and control groups were similar. (3.6 ± 1.7 vs 3.7 ± 1.8, *p* = 1.00), and their nutrition status both significantly improved during the last visit before discharged (2.6 ± 0.9 vs 2.9 ± 1.3).

**Conclusion:**

Probiotic supplementation in elderly hospitalized tube-fed patients significantly reduced stool frequency during antibiotic treatment. Improvements in stool form were observed only during the follow-up period. Nutritional status remained stable, with patients' nutritional needs adequately met throughout the study.

## Introduction

Antibiotics have significant benefits in treating many infectious diseases, but they often cause gastrointestinal (GI) side effects. Among them, antibiotic-associated diarrhea (AAD) is the most commonly experienced symptom by hospitalized patients, due to disruption of the GI microbiota and mucosal integrity, leading to overgrowth of pathogens and a metabolic imbalance. The incidence of AAD ranges 5% to 49%, and it can occur any time after antibiotics exposure [1]. About 10% ~ 25% of AAD cases were attributed to *Clostridium difficile* infection (CDI), which is commonly implicated with antibiotics including fluoroquinolones, clindamycin, penicillin, and third- or fourth-generation cephalosporin. *Clostridium difficile* (*C. difficile*) is a gram-positive, spore-forming bacillus that was first isolated in 1935 from the fecal flora of healthy neonates and was identified as the pathogen responsible for pseudomembranous colitis in 1978 [2–4].

*C. difficile-*associated diarrhea (CDAD) results in symptoms ranging from mild diarrhea to colitis, toxic megacolon, and death, and is the most common healthcare-associated diarrhea in developed countries. In a meta-analysis that reviewed 5496 hospitalized patients and the majority of studies was conducted in Asia, the frequency of CDAD among AAD was 20% (95% confidence interval [CI] 13.0–28.0) [5]. According to a study conducted at National Cheng Kung University Hospital, Taiwan, the proportion of hospitalized patients with CDAD was 42.6 cases per 100,000 patient-days, and 110.6 cases per 100,000 patient-days was in intensive care units (ICUs) [6]. In addition to antibiotics, advanced age and hospitalization are also risk factors for CDAD. A research on the outbreaks of *C. difficile* in Quebec found that individuals with an age over 65 years old had a ten-fold higher incidence rate when compared to younger individuals [7]. The incidence rate of CDAD among outpatient was 3%, while in hospitalized adults was ranged from 20 to 30% [8]. In a review that has also mentioned the complications of AAD could prolonged the length of stay, higher mortality rates, and increases medical cost. Moreover, the complications of CDI are much more severe than AAD that have higher rates of colectomies surgery and chances of readmissions [9]. It was reported that 61% of diarrhea in tube-fed patients was caused by medications, with *C. difficile* implicated in 17% of these cases [10]. According the above researches elderly hospitalized patients requiring tube feeding are particularly vulnerable to complications from antibiotic use.

Several studies have shown that specific strains of probiotics have various beneficial health effects, suggesting that providing probiotics may be helpful in control or prevention in AAD. Common probiotic strains associated with gastrointestinal health include *Lactobacillus rhamnosus* GG, *Bifidobacterium ssp*, *Streptococcus ssp*, *Yeast Saccharomyces boulardii*, and other *Lactobacillus* species such as* L. reuteri*, *L. rhamnosus*, *L. acidophilus*, and *L. casei*, and the used of multiple strains probiotics had a more significant effects in against CDAD when compared to using single strain probiotics [11]. As two studies have shown that the intervention with combination of *L.acidophilus* and *L. casei* showed improvements in decreasing incidence rates of AAD and CDAD among hospitalized patients. Furthermore, the suitable dosage of probiotics given is also crucial, as previous study has found that administering probiotic products with different dosage (5 × 10^10^ CFU vs 10^11^ CFU) to hospitalized elderlypatients significantly reduced the incidence rates of AAD and CDAD, while the highest dose had a better effect compared to low dose [12, 13]. Ouwehan et al. utilized a combination of *L. acidophilus*, *L. paracasei*, and *Bifidobacterium lactis* found that the both low-dose (4.17 × 10^9^ CFU) and high-dose (1.7 × 10^10^ CFU) in the probiotic group had significantly lower incidence rates of AAD and CDAD compared to the control group [14]. In a systematic review with meta-regression analysis concluded that administration of probiotics within 2 days of the first antibiotic dose reduces the risk of CDI by > 50% in hospitalized adults [15]. Although several studies indicated the beneficial effects of probiotics against the incidence of AAD in hospitalized patients, but the use of probiotics in elderly tube-fed patients treated with antibiotics was not investigated and it is rarely considered in the nutritional status of patients. Therefore, the aim of our study was to investigate the effects of probiotics in elderly hospitalized tube-fed patients with antibiotics use, assess the nutritional status of patients, and observe the prolonged effects after the end of antibiotics treatment.

### Methods

This study was conducted at the Taipei Medical University Hospital (TMUH). All research procedures performed in this trial were in strict accordance with a predefined protocol that was approved by all researchers and the local ethics committee.

The ethics committee approved the study protocol on 23 September 2020 and participants gave informed consent before participation. The certificate number of this study is N202008008.

### Subjects

Eligible patients were hospitalized, aged ≥ 65 years, being tube-fed, and had been prescribed antibiotics therapy for a minimum of 3 days and a maximum of 14 days. Exclusion criteria were the existence of a bowel disease such as a short bowel disease or inflammatory bowel disease, use of total parenteral nutrition, documented CDI within 3 months before enrollment, immunotherapy, and immunosuppressive disease such as hematological disease or acquired immunodeficiency syndrome (AIDS), antibiotics use within 30 days before enrollment, other probiotics use or participation in other clinical therapy.

### Study design

This is a single-center and case-controlled study. Each enrolled subject was matched with a contemporary control. The probiotic group received the first dose of the assigned intervention within 48 h of starting their prescribed antibiotics therapy and continued to use the product daily for an additional 7 days after completing their antibiotics were based on the previous researches [13, 14]. The control groups were those who obtained consent was greater than 48 h. We provided the commercial probiotics. The study product contained 10^9^ CFU of *L. plantarum*, *L. rhamnosus, L. acidophilus, B. lactis*, *S. thermophiles*, *L. casei* and *B. longum*. Intervention was administered twice daily (3 g per package, total 2 × 10^9^ CFU), approximately 2 h after antibiotics and meals. The investigators followed subjects via a weekly telephone call for four weeks after finishing the treatment of probiotics, to inquire about the stool form, adverse events and compliance with study product.

### Data collection

We collected the anthropometric data, laboratory data, medication, stool form, data from the medical record and assessed their nutrition status and nutrition intake by clinician or research nurse. The laboratory data included the complete blood count (CBC) to evaluate hematological status, albumin to evaluate nutrition status, creatinine (Cr) to assess renal function, C-reactive protein (CRP) as an indicator of systemic inflammation, and electrolyte levels, including sodium (Na) and potassium (K), to monitor fluid and electrolyte balance. Blood samples were performed according to hospital standard protocol. All patients received standard nutrition care process under a registered dietitian. Bristol Stool Form Scale form was used to assess the stool status. The Bristol Stool Form Scale can be used to monitor change in intestinal function, as such scales have been widely utilized in both clinical practice and research [16] (type 1–2 indicate constipation, type 3–4 are ideal stools as they are easier to pass and type 5–7 may indicate diarrhea and urgency). Nutrition risk screening (NRS) 2002 score is used to assess the nutritional risk which is a sum of the total of the nutritional score, severity of disease score, and the age adjustment score that may provide a clear and clarify statement in malnutrition, by giving a total number of points ranges from 0 to 7 [17]. We defined the score 0 as well nourished, score 1–2 as mild malnutrition risk, score 3–4 as moderate malnutrition risk and score ≥ 5 as high malnutrition risk. Subjects were supplied with diary cards and instructed by the physician to record any diarrhea and number of liquid stools per day during experimental period.

### Statistical methods

Data were expressed as MEAN ± SD. Two-tailed χ^2^ test or a Fisher’s exact test to compare between the groups. Continuous data was analyzed by nonparametric tests (Wilcoxon’s rank sum or Mann–Whitney U test). The correlation between variables in the study groups was used the Spearman’s correlation. *p* < 0.05 was considered statistically significant. SPSS version 26.0 was used to perform statistical analysis. The calculated statistical power for the independent samples t-test, assuming an effect size of 0.8 and a sample size of 11 in each group at a significance level of 0.05, was found to be 0.51.

## Results

**Fig.1:**
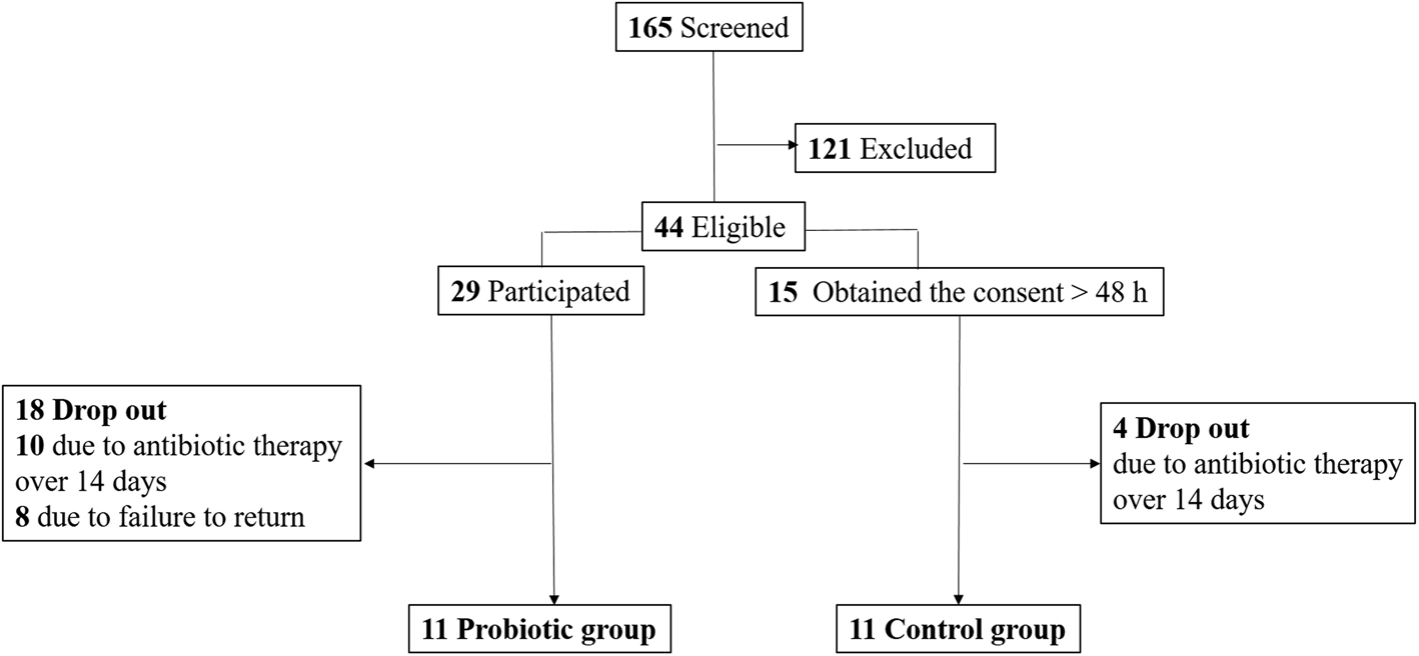
Participants enrollment through the study

In total, 165 patients were screened, and 121 patients were excluded. Total 44 patients were eligible to participate in the study, and 29 patients signed the consent form within 48 h, and these served as probiotic group. The other 15 patients served as control group due to the time to obtain consent was greater than 48 h. Both groups had patient who failed to complete the study either due to the use of antibiotics for more than 14 days or personal reasons. 11 patients completed the study in both group (Fig. 1). Average ages of the probiotic and control groups were 80.0 ± 10.2 years and 83.1 ± 10.3 years, respectively. The initial NRS 2002 score of the probiotic and control groups were 3.6 ± 1.7 and 3.7 ± 1.8, respectively, and their nutrition status both significantly improved during the last visit before discharge (2.6 ± 0.9 and 2.9 ± 1.3, respectively). There were no significant differences in baseline characteristics between the two groups, except the potassium level. Nevertheless, the potassium levels in both groups were within normal range (Table 1).

**Table 1:** Basic characteristics of the study groups

	**Control (*n* = 11)**	**Probiotic (*n* = 11)**
Male sex (%)	63.6%	54.5%
BMI (kg/m^2^)	22.1 ± 4.2	21.9 ± 2.6
Age (yr)	83.1 ± 10.3	80.0 ± 10.2
Length of hospitalization (d)	14.9 ± 6.9	12.1 ± 6.4
NRS score_initial	3.7 ± 1.8	3.6 ± 1.7
NRS score_discharge	2.9 ± 1.3^*^	2.6 ± 0.9^*^
ICU admit (%)	27.3%	45.5%
Duration of antibiotics therapy (d)	10.8 ± 2.9	10.3 ± 4.4
Numbers of antibiotics	1.9 ± 0.8	2.2 ± 1.0
Antibiotics type, n (%)		
Frequently associated CDI		
Broad-spectrum penicillin	6 (54.5)	6 (54.5)
Lincosamide	1 (9.1)	0 (0.0)
2nd-generation cephalosporin	3 (27.3)	4 (36.4)
3rd-generation cephalosporin	0 (0.0)	7 (63.6)
4th-generation cephalosporin	0 (0.0)	2 (18.2)
Occasionally associated CDI		
1st-generation cephalosporin	2 (18.2)	0 (0.0)
Macrolide	3 (27.3)	1 (9.1)
Penicillinase-sensitivity penicillin	1 (9.1)	1 (9.1)
Rarely associated CDI		
Aminoglycoside	2 (18.2)	2 (18.2)
Vancomycin	3 (27.3)	0 (0.0)
Laboratory data		
Hemoglobin (g/dL)	10.1 ± 1.7	11.6 ± 1.9
WBC (10^3^ /uL)	8.9 ± 3.9	10.9 ± 6.6
% of Neutrophil	70.7 ± 14.8	75.9 ± 12.0
% of Lymphocyte	18.3 ± 11.0	13.7 ± 9.3
Cr (mg/dL)	1.3 ± 1.7	0.9 ± 0.6
Albumin (g/dL)	3.2 ± 0.7	3.5 ± 0.8
CRP (mg/dL)	5.4 ± 4.1	6.3 ± 8.7
Na (mEq/L)	136.0 ± 8.3	136.7 ± 10.9
K (mEq/L)	4.6 ± 1.0	3.8 ± 0.4^§^
CDI occurrence rate	9.0%	0.0%

*BMI* body mass index, *ICU* intensive care unit, *NRS* nutrition screening tools, *WBC* white blood count, *BUN* blood urea nitrogen, *Cr* creatinine, *CRP* C-reactive protein, *CDI* Clostridium difficile infection

^*^p < 0.05 when compared with the NRS socre_initial within group

^§^*p* < 0.05 showed significant difference between group

**Fig. 2:**
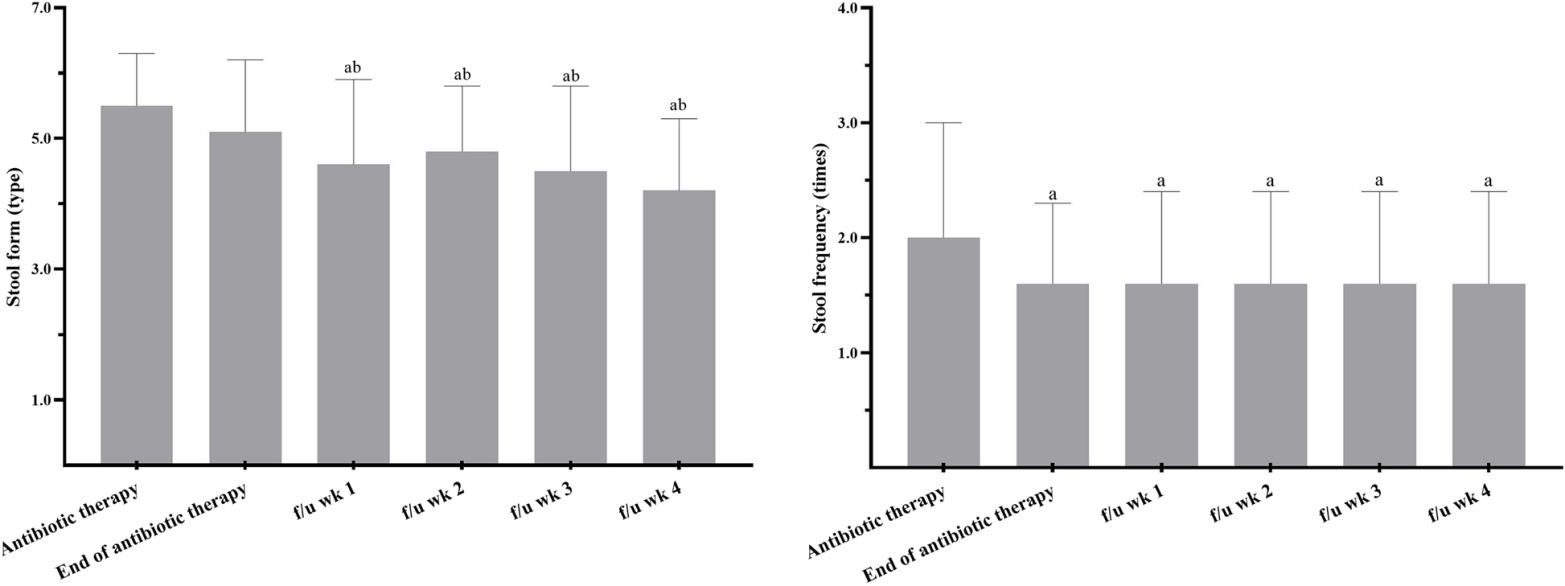
The stool form (left) and stool frequency (right) in probiotic group. Stool form: type 1–2 indicate constipation, 3–4 are ideal stools as they are easier to pass and 5–7 may indicate diarrhea and urgency. f/u, follow up; wk, week. ^a^*p* < 0.05 when compared with the beginning of antibiotics therapy within group by using Wilcoxon Signed Ranks Test. ^b^*p* < 0.05 when compared with the end of the antibiotics therapy within group by using Wilcoxon Signed Ranks Test

The average number of days that probiotics were given was 16.0 ± 2.8 days. Compliance with the study product was 100%, as there were no returned study products, and they were consumed according to the patients' daily cards. The mean stool forms at the beginning of the antibiotics therapy in the probiotic and control group were 5.6 ± 1.4 and 5.5 ± 0.8, with frequencies of 2.0 ± 1.0 and 2.7 ± 2.1, respectively, and there were no significant differences between these two groups. In the probiotic group, no significant difference was found in stool form between the beginning and end of antibiotics therapy (5.5 ± 0.8 vs. 5.1 ± 1.1, *p* = 0.21), but the frequency significantly decreased (2.0 ± 0.7 vs. 1.6 ± 0.7, *p* = 0.05), whereas in the control group, although the stool form between beginning and end of antibiotics therapy significantly improved (5.6 ± 1.4 vs. 4.5 ± 1.4, *p* = 0.01), the frequency did not significantly differ (2.7 ± 2.1 vs. 2.4 ± 1.5, *p* = 0.10). During the follow-up period of the probiotic group (a total of 4 weeks), the stool form significantly differed compared to both the beginning and end of antibiotics therapy. The frequency during the follow-up period also significantly decreased compared to the beginning of antibiotics therapy (2.0 ± 1.0 vs. 1.6 ± 0.8, *p* = 0.02), but no difference when compared to the end of antibiotics therapy (Fig. 2).

Both the probiotic and control groups had similar estimated nutritional requirements. The feeding formula was a standard formula without the addition of fiber in both groups. The estimated calorie requirement for the probiotic and control groups were 1455.6 ± 113.0 kcal and 1512.5 ± 155.3 kcal, respectively, and the actual intake percentages of calorie requirements were 98.1% and 91.2%. The estimated protein requirement for the probiotic and control groups 68.5 ± 11.5 g/d and 67.9 ± 9.0 g/d and the actual intake percentages of protein requirements were 91.7% and 88.1%. The actual calorie and protein intake showed no significant differences between the groups (Table 2).

**Table 2:** The nutrition requirement and actual intake in study groups (2nd visit)

	**Control**	**Probiotic**
Estimate calorie requirement (kcal)	1512.5 ± 155.3	1455.6 ± 113.0
Actual calorie intake (kcal)	1380.1 ± 289.1	1428.6 ± 180.8
Estimate protein requirement (g)	67.9 ± 9.0	68.5 ± 11.5
Actual protein intake (g)	59.8 ± 15.7	62.8 ± 13.4
Data expressed as MEAN ± SD		

Variables such as ICU admission, duration of antibiotics therapy, and numbers of antibiotics used were assessed using Spearman’s correlation. These variables did not show any correlation in the probiotic group. However, in the control group, the stool form at the beginning of antibiotics therapy was moderately correlated with ICU admission (*r* = 0.67, *p* < 0.001). The stool frequency at the beginning of antibiotics therapy and the initial NRS 2002 score were highly correlated with ICU admission (*r* = 0.84, *p* < 0.001; *r* = 0.81, *p* < 0.001) (Table 3).

**Table 3:** The correlation between variables in study groups

		**Control**		**Probiotic**	
	**Variables**	**R**	***p***	**R**	***p***
ICU admit	Length of hospitalization	0.23	0.54	0.33	0.32
	Duration of antibiotics therapy	0.30	0.38	0.21	0.54
	Kinds of antibiotics	0.55	0.08	0.22	0.51
	Stool form_antibiotics therapy	0.67^*^	0.00	−0.06	0.85
	Stool frequency_antibiotics therapy	0.84^*^	0.00	0.16	0.65
	Stool form_ end of antibiotics therapy	−0.27	0.43	−0.4	0.22
	Stool frequency_end of antibiotics therapy	0.49	0.13	0.00	1.00
	NRS score_initial	0.81^*^	0.00	0.38	0.24
	NRS score_discharge	0.43	0.19	−0.14	0.68
Duration of antibiotics therapy	Stool form_antibiotics therapy	0.20	0.56	0.31	0.35
	Stool frequency_antibiotics therapy	0.04	0.90	0.50	0.11
	Stool form_ end of antibiotics therapy	0.13	0.70	0.01	0.98
	Stool frequency_end of antibiotics therapy	0.12	0.71	0.08	0.82
Numbers of antibiotics	Stool form_antibiotics therapy	0.52	0.10	−0.22	0.51
	Stool frequency_antibiotics therapy	0.52	0.10	0.16	0.64
	Stool form_ end of antibiotics therapy	−0.15	0.67	0.19	0.59
	Stool frequency_end of antibiotics therapy	0.29	0.39	0.08	0.81

^*^p < 0.05 indicates the correlation is significant by using Spearman’s correlation

## Discussion

Our study is the first study that concern the nutritional status among the patient that who are at high risk of developing CDI. We found that the initial NRS 2002 score in both groups were moderate malnutrition risk (score ≥ 3). Until the last visit before discharge, the scores in both groups had decreased significantly, indicating an improvement in their nutritional status. In a retrospective study which was performed in France, it was found that populations with malnutrition were correlated with CDI. But its definition was based on body weight loss, lower BMI, low calorie intake, with a low albumin and high CRP levels [18], and such definition may not clarify the severity of malnutrition. Our study used the NRS 2002 score to assess the nutrition status, which has a clarified definition. The NRS 2002 score can predict the validity the clinical outcome such as to the improvement of patients who identified to be at risk. For adult patients in hospital, the NRS 2002 is recommended and if score ≥ 3 need to generate a nutrition plan [19]. We found that the patients in both groups were moderate malnutrition risk as both have a score over 3. Till the last visit in hospital, their nutritional status improved significantly. Furthermore, both inadequate or excessive feeding may be harmful in adult hospitalized patients, hence consultation from dietitians or other experts on feed prescription is crucial [20]. According to European Society for Clinical Nutrition and Metabolism (ESPEN) guideline, the calorie delivery should be subsequently increased up to 80–100% of the measured energy expenditure after 3 days in the ICU [21]. In this study, patients were met 80% of their nutrition requirement on the second visit in the probiotic and control groups no matter admit to ICU or not. Retrospective studies showed that fiber supplements can help lessen post-feeding diarrhea in hospitalized patients receiving enteral nutrition and the formula and the volume provided are factors that could influence whether patients have diarrhea [22, 23]. The feeding formula in this study was a standard formula without the addition of fiber in both groups. The consistency of enteral feeding formula and comparable nutritional intake across groups reduces the likelihood of dietary intake influencing the observed outcomes, ensuring that the effects can be attributed to the probiotic intervention.

The use of antibiotics is an effective option when it comes to treating infectious diseases, but inappropriate use of broad-spectrum antibiotics, and the emergence of drug-resistant bacteria, has raised significant clinical concerns, such as CDAD which is one of the most frequent consequences led by inappropriate use of antibiotics treatment [24]. A system review indicated the used of probiotic could reduce the risk of CDI by 60.5% in both adults and children treated with antibiotics in inpatient and outpatient [25]. Additionally, probiotics have been suggested to prevent or treat CDI through various potential mechanisms proposed by Mills et al. A) Probiotics could compete the favorable environment, inhibiting the growth of vegetative *C. difficile*. B) Decreased metabolism of primary to secondary bile acids by 7α-dehydroxylase promotes germination of *C. difficile* spores, which can be counteracted by administering bacteria with such activity. C) The *C. difficile* toxins A and B, responsible for the symptoms of the disease, can be neutralized through the secretion of inhibitory compounds. D) Probiotics secrete bacteroicins that may inhibit *C. difficile* by altering pH, increasing mucosal IgA levels, or enhancing mucin production [26–29]. The characteristics of our study groups were over 50% of male gender, taking 2 or more antibiotics, the length of hospitalization were about 15 days and the using days of probiotic days was 16.0 ± 2.8 days in probiotic group. Although our study showed no significant difference on reducing CDI. We had the similar characteristics with a meta-analysis study which suggested that probiotic may be useful and safe in preventing CDI. The characteristics of these subjects were male (over 50%), taking 2 or more antibiotics, the median length of hospital stays was 7 days (4–15 days), the median duration of antibiotics therapy was 10 days (7–14 days) and the median using days of probiotic was 15 days (11–21 days) [30].

The Bristol Stool Scale demonstrated the validity and reliability in diarrhea-predominant irritable bowel syndrome [31]. In our study, we found that the stool form in the probiotic group much closer to the ideal form (type 3 and 4) during follow-up compared to the beginning or end of antibiotics therapy. This was not observed in the control group, even though we used a lower dosage (2 × 10^9^ CFU) than in previous studies that also use this scale to assess the stool form. For example, a triple-blind randomized controlled trial (RCT) used capsule that containing *L. acidophilus*, *L.paracasei* and *B. lactis* found that liquid stool was less common in both the low-dose group (4.17 × 10^9^ CFU) and high-dose (1.70 × 10^10^ CFU) probiotic groups [14]. Another double-blind RCT using fermented milk containing *L. acidophilus* CL1285® and *L. casei* observed a lower incidence of loose stool in the high dose group (10^11^ CFU) among individuals aged 50–70 [13]. However, a dose of 5 × 10^10^ CFU of the same product did not show any significant decrease in the incidence or adverse effects of CDAD [12].

A growing body of evidence indicates that critical illness and widespread of antibiotics use, resulted in gut dysbiosis in ICU patients which is associated with higher rates of infections, sepsis, and multiple organ dysfunction syndrome [32]. We found the positive correlation between stool form and frequency during antibiotics therapy and ICU admit in control group whereas not in probiotic group, indicate that the use of probiotics could have potential benefits of preventing diarrhea in ICU patients. A case controlled trial have also demonstrated that fermented milk containing 10 billion of *L.casei* could be provided to ICU patients without no serious adverse effects such as insertion of rectal tube for diarrheal control, emesis, requirement for parenteral nutrition, and need for surgical management of GI tract, suggesting its safeness and stability used in ICU patients [33].

The reasons for the inconsistency between our study and previous research results may include that some strains of our probiotics are not commonly used for CDI, such as *L.* species and *B. longum* are more commonly investigated in studies of inflammatory bowel disease and colitis [34, 35], while *Streptococcus thermophiles* is typically used for reducing symptoms of lactose intolerance [36]. Moreover, the composition of the gut microbiota might differ due to factors such as age, diet, lifestyle, antibiotics, probiotics, or geographical variation, potentially leading to different outcomes compared to previous studies [37–39]. There are some limitations in our study. Firstly, the small sample size due to the reduced number of hospitalized patients during the COVID-19 pandemic, and the inability to contact and obtain informed consent from family members due to visiting restrictions, resulting in a lower enrollment rate. Secondly, the incomplete data from the control group makes it challenging to compare with the experimental group. Consequently, tracking stool form and frequency during the follow-up period becomes difficult, resulting in incomplete data that cannot be analyzed. Lastly, we didn’t design the comparison between different compositions or dosages of the probiotic strains.

In conclusion, this study demonstrated that probiotic supplementation in elderly tube-fed patients using antibiotics significantly reduced stool frequency during antibiotic treatment, though no significant changes were observed in stool form during this period. Notably, improvements in stool form were observed only during the follow-up period after antibiotic use. Nutritional status remained stable throughout the study, with patients’ nutritional needs adequately met. Further studies are required to explore the prolonged effects of probiotics post-antibiotic therapy and their role in supporting overall nutritional outcomes.

## Data Availability

The datasets used and/or analzsed during the current study are available from the corresponding author on reasonable request.

## Abbreviations

GI: Gastrointestinal
AAD: Antibiotic-associated diarrhea;
*C. difficile*: *Clostridium difficile*
CDI: *Clostridium difficile* Infection
CDAD: *C. difficile-*Associated diarrhea
ICU: Intensive care units
AIDS: Acquired immunodeficiency syndrome
NRS 2002: Nutrition risk screening 2002
BMI: Body mass index
RCT: Randomized controlled trial
